# Molecular Manipulation of MicroRNA397 Abundance Influences the Development and Salt Stress Response of *Arabidopsis thaliana*

**DOI:** 10.3390/ijms21217879

**Published:** 2020-10-23

**Authors:** Duc Quan Nguyen, Christopher W. Brown, Joseph L. Pegler, Andrew L. Eamens, Christopher P. L. Grof

**Affiliations:** Centre for Plant Science, School of Environmental and Life Sciences, University of Newcastle, Callaghan, NSW 2308, Australia; DucQuan.Nguyen@uon.edu.au (D.Q.N.); christopher.brown@uon.edu.au (C.W.B.); joseph.pegler@newcastle.edu.au (J.L.P.)

**Keywords:** *Arabidopsis thaliana*, *Setaria viridis*, microRNA397 (miR397), *LACCASE* (*LAC*), lignin, salt stress, gene expression regulation, RT-qPCR

## Abstract

*Arabidopsis thaliana* (*Arabidopsis*) has been used extensively as a heterologous system for molecular manipulation to genetically characterize both dicotyledonous and monocotyledonous plant species. Here, we report on *Arabidopsis* transformant lines molecularly manipulated to over-accumulate the small regulatory RNA microRNA397 (miR397) from the emerging C_4_ monocotyledonous grass model species *Setaria viridis* (*S. viridis*). The generated transformant lines, termed *SvMIR397* plants, displayed a range of developmental phenotypes that ranged from a mild, wild-type-like phenotype, to a severe, full dwarfism phenotype. Reverse transcriptase quantitative polymerase chain reaction (RT-qPCR)-based profiling of the *SvMIR397* transformant population revealed a strong correlation between the degree of miR397 over-accumulation, repressed *LACCASE* (*LAC*) target gene expression, reduced lignin content, and the severity of the developmental phenotype displayed by *SvMIR397* transformants. Further, exposure of *SvMIR397* transformants to a 7-day regime of salt stress revealed the *SvMIR397* transformant lines to be more sensitive to the imposed stress than were wild-type *Arabidopsis* plants. Taken together, the findings reported here via the use of *Arabidopsis* as a heterologous system show that the *S. viridis* miR397 small regulatory RNA is able to repress the expression of three *Arabidopsis LAC* genes which led to reduced lignin content and increased salt stress sensitivity.

## 1. Introduction

Lignin is the third most abundant heteropolymer in plant secondary cell walls [1,2] and is primarily composed of three subunits, the *p*-hydroxyphenyl (H), guaiacyl (G), and syringyl (S) subunits, derived from the three monolignols, *p*-coumaryl, coniferyl, and sinapyl alcohol, respectively [3,4]. The deposition of lignin is usually initiated during the formation of secondary cell walls after the completion of cell elongation. During the lignification process, monolignols are synthesized in the cytosol via a sequential set of chemical reactions catalyzed by 10 key lignin biosynthetic enzymes, and after their production, the monolignols are transported to the apoplasm where they are embedded in the secondary cell wall by an oxidative polymerization process catalyzed by LACCASE (LAC) enzymes [2,4]. In vascular plants, lignin is covalently linked to cellulose and hemicellulose to confer shape, high mechanical strength, and the hydrophobicity of plant cell walls, thereby playing a crucial role in the erect growth of a plant, nutrient transport, and protection against specific abiotic and biotic stresses [2,5].

Soil salinity is a major environmental problem worldwide that affects the agricultural productivity (plant yield and biomass) and sustainability of approximately 20% of the world’s useable agricultural land [6,7]. Increased salinization of arable land is the result of excessive salt accumulation in the soil through either natural causes, such as coastal flooding and high evapotranspiration rates, or by human-related activities such as poor irrigation practices [6,7]. Soil salinity is toxic to most crop species because it inhibits the conventional growth and development of these species by interfering with numerous molecular and physiological processes. Namely, the excessive accumulation of salt in plant tissues can rapidly lead to high osmotic pressure, loss of turgor pressure, ionic (sodium, Na^+^, and chloride, Cl^−^, ions) toxicity, and reactive oxygen species (ROS)-derived oxidative stress [8,9,10]. This results in the closure of stomata, cessation of cell growth and expansion, reduction in the rate of photosynthesis, reduced carbon assimilation, and the uptake of water and nutrients, which together promote the premature transition from vegetative to reproductive development and the early onset of senescence [11,12]. Salt stress can also indirectly and broadly affect the physicochemical properties of plant cell walls by altering the expression of cell wall biosynthesis genes and/or the functional modification of cell wall enzyme activity, and hence, the composition of cell wall components. For instance, salt stress severely decreased the amount of cellulose in the roots of rice (*Oryza sativa*) and cotton (*Gossypium hirsutum*) plants [13,14], and altered the structural arrangement of the majority of the cellulose microfibril network (91% of the investigated cells) in sorghum (*Sorghum bicolor*) longitudinal cell walls, forming a close-meshed network instead of a parallel array [15].

In response to salt stress, plants have developed various elaborate mechanisms involved in the manipulation of plant morphology, physiology, and metabolism to survive its potentially detrimental effects [16,17]. At the molecular level, plant responses to salt stress have been determined to be controlled by various stress-response genes, including those loci that encode for microRNA (miRNAs) small RNAs (sRNAs) [18,19,20,21]. Plant miRNAs are a class of small regulatory RNA, predominantly 21 nucleotides (21-nt) in length, that control gene expression at the posttranscriptional level across all phases of plant growth and development as well as directing the molecular response of a plant to specific abiotic or biotic stress challenges [22,23,24]. To date, numerous studies have investigated the involvement of miRNA-directed responses to salt stress across a wide range of plant species. For example, Pegler and colleagues [19,20] have profiled the salt stress-responsive miRNA landscapes of the experimental model plant species, *Arabidopsis thaliana* (*Arabidopsis*) and *Setaria viridis* (*S. viridis*), to reveal that many miRNAs differentially accumulate in response to salt stress challenge. Among the miRNAs identified in *Arabidopsis* and *S. viridis* to be responsive to salt stress was the miR397 sRNA. At the posttranscriptional level, miR397 has been demonstrated to regulate the expression of a number of *LAC* gene family members in both monocotyledonous and dicotyledonous plant species [25,26]. The encoded LAC proteins are members of the multi-copper oxidase superfamily that are responsible for catalyzing the oxidative polymerization of lignin subunits in secondary cell walls [2,4]. In a number of plant species, the overexpression of miR397 precursor sequences, leading to over-accumulation of the miR397 sRNA, has been demonstrated to result in decreased miR397-targeted *LAC* transcript expression; reduced lignin subunit abundance (S and G subunits); and morphological abnormalities, including semi- and full dwarfism, as well as the development of deformed and/or collapsed vascular tissues [26,27,28,29]. However, to date, the exact role that the miR397 sRNA plays in the posttranscriptional regulation of *LAC* gene expression in *S. viridis* remains to be investigated.

Here, we provide strong molecular evidence that the miR397 sRNA of *S. viridis* (*SvMIR397*) is capable of posttranscriptionally regulating the expression of the *LAC* gene family members, *LAC2*, *LAC4*, and *LAC17*, when heterologously overexpressed in *Arabidopsis*. More specifically, when the miR397 sRNA from either *Arabidopsis* or *S. viridis* was overexpressed in *Arabidopsis*, termed *AtMIR397* and *SvMIR397* transformants, respectively, a significant decrease in *LAC2*, *LAC4*, and *LAC17* target gene expression and total lignin content was detected. In addition, we assessed the molecular and phenotypic responses of *AtMIR397* and *SvMIR397* transformants to salt stress challenge for comparison with those displayed by unmodified wild-type *Arabidopsis* plants (ecotype Columbia-0 (Col-0)). The *AtMIR397* and *SvMIR397* transformants were determined to be more sensitive than wild-type *Arabidopsis* plants to salt stress. Furthermore, RT-qPCR demonstrated that miR397 abundance was induced to a greater degree by the imposed stress in the *AtMIR397* and *SvMIR397* transformants, and accordingly, *LAC2*, *LAC4*, and *LAC17* expression was reduced to a greater extent in these two transformant populations than it was in wild-type *Arabidopsis*. Therefore, when taken together, the results presented here indicate that the miR397 sRNA and/or its regulated *LAC* target genes, potentially play a central and conserved role in the response of a plant to salt stress challenge.

## 2. Results

### 2.1. Phenotypic and Physiological Assessment of Arabidopsis thaliana Plants Molecularly Manipulated to Over-Accumulate the MicroRNA397 Small RNA

**Figure 1:**
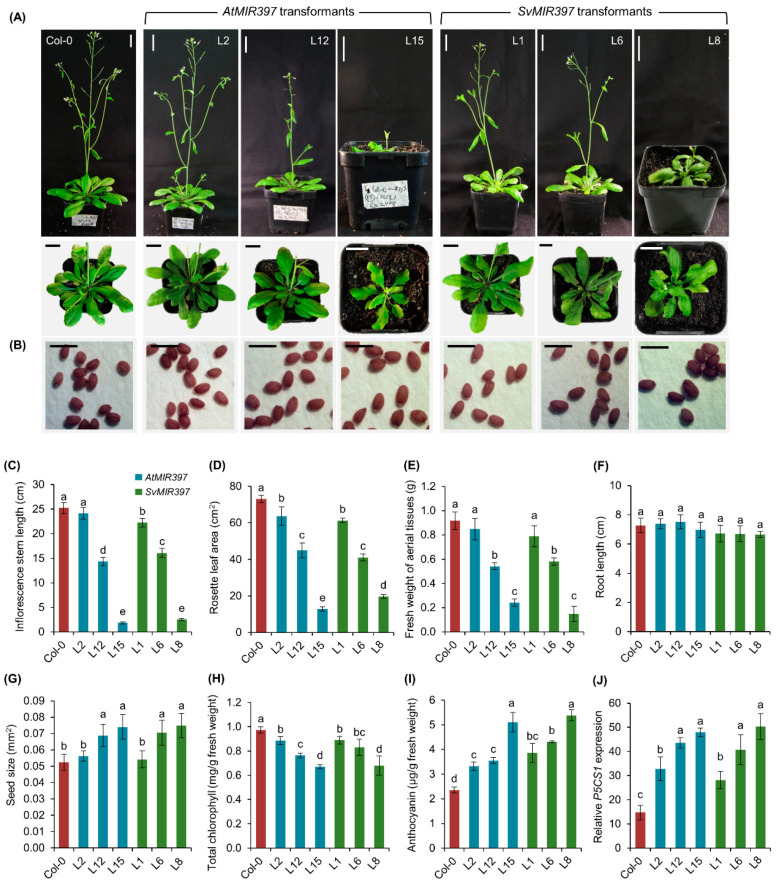
Phenotypic and physiological analyses of the *AtMIR397* and *SvMIR397* transformant populations. (**A**) Phenotypes displayed by the phenotypic grouping representatives, including *AtMIR397* transformants L2 (Group—1), L12 (Group—2), and L15 (Group—3), and the *SvMIR397* transformants L1 (Group—1), L6 (Group—2), and L8 (Group—3). Bar = 2.0 cm. (**B**) Seed morphology of the phenotypic grouping representatives, including transformants *AtMIR397* L2, L12, and L15, and *SvMIR397* transformants L1, L6, and L8. Bar = 500 µm. Phenotypic comparisons of the (**C**) inflorescence stem length (cm), (**D**) rosette area (cm^2^), (**E**) aerial tissue fresh weight (g), (**F**) primary root length (cm), and (**G**) seed size (mm^2^) of Columbia-0 (Col-0) plants and the six assessed transformant lines. (**H**) Total chlorophyll (mg/g fresh weight) and (**I**) anthocyanin content (µg/g fresh weight) in the rosette leaves of Col-0 plants and the six assessed transformant lines. (**J**) RT-qPCR assessment of the expression of the *Arabidopsis* stress marker gene, *P5CS1*, in Col-0 plants and the six assessed transformant lines. Statistical data were analyzed using one-way ANOVA and Tukey’s post hoc tests. The statistically significant differences are indicated by a different letter (*p-*value < 0.05) above each of the different colored columns of each presented histogram.

*Arabidopsis thaliana* (ecotype Columbia-0 (Col-0)) plants molecularly modified to overexpress the *PRE-MIR397B* precursor transcript of either *Arabidopsis* or *S. viridis* displayed a range of developmental phenotypes of differing severity. More specifically, the *AtMIR397* and *SvMIR397* transformant populations were placed into one of three groupings depending on the severity of the developmental phenotype expressed by each transformant line, including Group—1, the expression of a mild or wild-type-like phenotype; Group—2, the expression of a moderate phenotype characterized by semi-dwarfism; and Group—3, the expression of a severe phenotype as characterized by this population of plants displaying full dwarfism (Figure 1A). In addition to the initial visual analysis to place each obtained transformant line into one of the three phenotypic groupings, the (1) length of the primary inflorescence, (2) rosette area, (3) fresh weight of aerial tissues, and (4) length of the primary root for each of the selected transformants were all recorded to ensure that each plant line had been placed into the appropriate phenotypic group.

For the *AtMIR397* transformant population (*n* = 70), the length of the inflorescence stem varied from 24.1 cm for the Group—1 representative, transformant line 2 (L2), to 1.8 cm for transformant *AtMIR397* L15, the Group—3 representative (Figure 1A,C). Transformants *AtMIR397* L2 and L15 were also determined to have the largest (63.7 cm^2^) and smallest (12.8 cm^2^) rosette areas (Figure 1D), respectively, and therefore accordingly, *AtMIR397* L2 returned the heaviest (0.85 g), and *AtMIR397* L15 the lightest (0.24 g) fresh weight (Figure 1E). The transformant *AtMIR397* L12 was selected as the Group—2 or moderate (semi-dwarf) phenotype group representative. This transformant line developed a primary inflorescence of 14.3 cm in length that was almost devoid of branching (Figure 1A). Due to the *AtMIR397* L12 plant producing fewer rosette leaves than those of wild-type *Arabidopsis* plants at an equivalent stage of development (Figure 1B), the rosette area of the Group—2 representative *AtMIR397* L12 was only 44.9 cm^2^, and was determined to have a fresh weight of 0.54 g; a 40.9% reduction in fresh weight compared to that of Col-0 plants.

A similar set of analyses were conducted on the *SvMIR397* transformant population (*n* = 40). For the *Arabidopsis* plants molecularly modified to overexpress the introduced *S. viridis PRE-MIR397B* precursor transcript, transformant lines L1, L6, and L8 were selected as representatives of the mild (Group—1), moderate (Group—2), and severe (Group—3) phenotypic groupings, respectively (Figure 1A). The *SvMIR397* transformant L1 exhibited the longest stem length at 22.3 cm (Figure 1C) and the largest rosette area at 61.3 cm^2^ (Figure 1D). Therefore, transformant line *SvMIR397* L1 was also determined to have the heaviest fresh weight of the assessed transformant population at 0.79 g (Figure 1E). In direct contrast to the *SvMIR397* Group—1 representative, the Group—3 representative, transformant *SvMIR397* L8, was determined to have the lowest fresh weight value (0.15 g), the shortest inflorescence stem length (2.5 cm), and the smallest rosette leaf area (19.7 cm^2^) among the 40 *SvMIR397* transformant lines analyzed (Figure 1A,C–E).

The size of seeds harvested from each *AtMIR397* and *SvMIR397* transformant line was also measured. This assessment revealed that the seeds from *AtMIR397* transformants L12 (Group—2) and L15 (Group—3) were increased in size by 31.5 and 41.3%, respectively, compared to the seeds harvested from wild-type *Arabidopsis* plants (Figure 1B,G). Unsurprisingly, the size of the seeds generated by the *AtMIR397* Group—1 representative transformant L2, which displayed a wild-type-like phenotype, was determined to be of an equivalent size to those of Col-0 plants (Figure 1B,G). This analysis also returned highly similar results for the *SvMIR397* transformant population. That is, *SvMIR397* transformants L6 (Group—2) and L8 (Group—3) developed seeds that were 34.7 and 42.9% larger than those of Col-0 plants, respectively, while the Group—1 wild-type-like plant *SvMIR397* L1 generated seeds equivalent in size to those of wild-type *Arabidopsis* (Figure 1B,G). Considering the range of developmental phenotypes documented for the aerial tissues of *AtMIR397* and *SvMIR397* transformants, it was highly surprising to determine that the primary root length of all transformant lines, regardless of their phenotypic group classification, remained equivalent in length to the primary root length of unmodified Col-0 plants (Figure 1F).

The range of developmental phenotypes expressed by the *AtMIR397* and *SvMIR397* transformant populations strongly indicated that the generated plant lines may have been perceiving a degree of stress even when these plant lines were germinated and cultivated under standard *Arabidopsis* growth conditions. Therefore, the total chlorophyll (chlorophyll *a* and *b* content combined) and anthocyanin (a natural plant antioxidant) content was also measured for comparison to wild-type plants [30]. Figure 1H shows that the overexpression of the *Arabidopsis* and *S. viridis PRE-MIR397B* precursor sequence reduced the total chlorophyll content in all transformant lines compared to Col-0 plants. More specifically, compared to wild-type *Arabidopsis*, total chlorophyll was reduced by 9.0, 21.5, and 31.0% in *AtMIR397* transformants L2, L12, and L15, respectively, and by 8.6, 14.8, and 30.1% in the *SvMIR397* transformants L1, L6, and L8, respectively (Figure 1H). In contrast to the chlorophyll content of *AtMIR397* and *SvMIR397* transformants, the accumulation of anthocyanin was determined to increase in accordance with the severity of the displayed phenotype (Figure 1I). Namely, compared to non-modified Col-0 plants, the accumulation of anthocyanin was increased by 41.0 and 63.6% in the Group—1 representatives (*AtMIR397* L2 and *SvMIR397* L1, respectively), further elevated to 50.8 and 82.9% in the Group—2 members (*AtMIR397* L12 and *SvMIR397* L6, respectively), and elevated again by 116.5 and 128.3% to its maximum abundance in the Group—3 transformants, *AtMIR397* L15 and *SvMIR397* L8, respectively. Reduced total chlorophyll, in parallel with increased anthocyanin abundance, strongly suggested that indeed the *AtMIR397* and *SvMIR397* transformants were “stressed” even when cultivated in a standard *Arabidopsis* growth environment. Therefore, we next used a standard RT-qPCR approach to assess the expression of a well-known marker gene in *Arabidopsis* for plant stress responses, *DELTA-1-PYRROLINE-5-CARBOXYLATE SYNTHETASE1* (*P5CS1*; *AT2G39800*). RT-qPCR revealed significantly enhanced *P5CS1* expression in all six *MIR397* transformant lines assessed (Figure 1J). Unsurprisingly, the greatest enhancement to *P5CS1* transcript abundance was observed in the two transformants selected to represent the severe developmental phenotype grouping (Group—3) of each transformant population with *P5CS1* transcript abundance upregulated by 3.3- and 3.4-fold in lines *AtMIR397* L15 and *SvMIR397* L8, respectively (Figure 1J).

### 2.2. Anatomical Assessment of Arabidopsis thaliana Plants Molecularly Manipulated to Over-Accumulate the MicroRNA397 Small RNA

**Figure 2:**
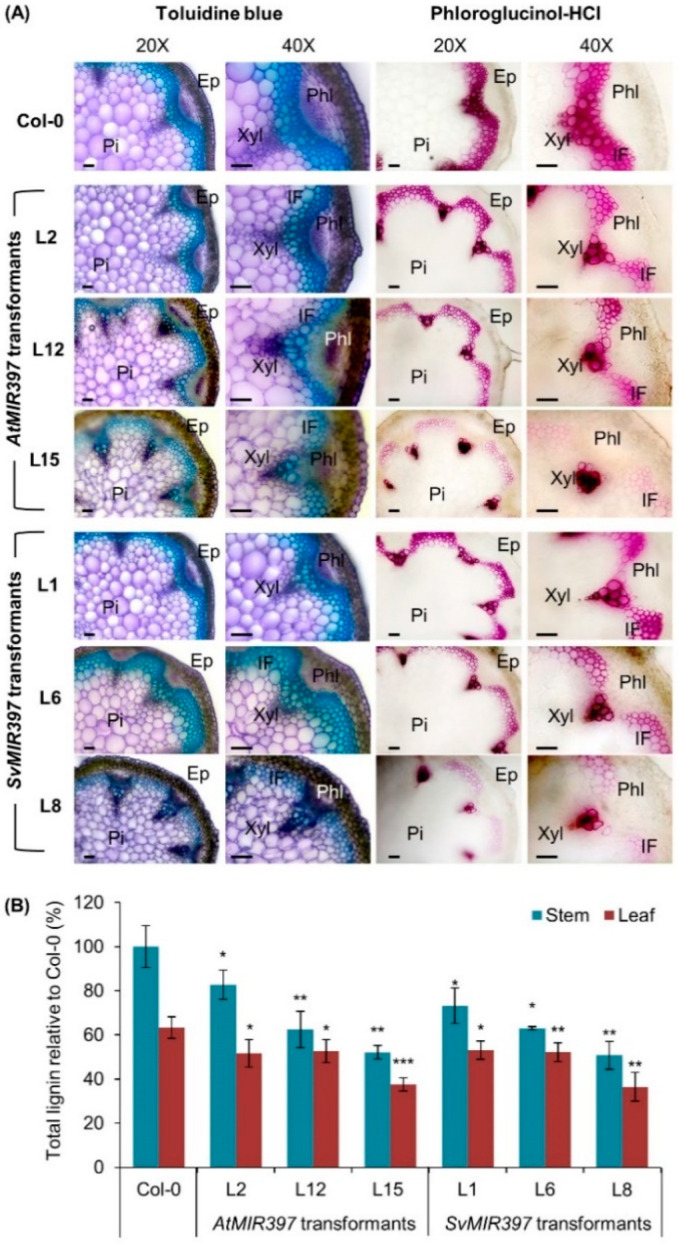
Visual and total lignin quantification of *AtMIR397* and *SvMIR397* transformant lines. (**A**) Cross-sections of the inflorescence stem of Col-0 plants transformed to over-accumulate the miR397 small RNA (sRNA). Toluidine blue staining was conducted to assist the analysis of anatomical features for the cross-sections of the primary inflorescence stem of *Arabidopsis* transformants. Phloroglucinol-HCl staining was performed to investigate the lignin content, and the location of its deposition, in the primary inflorescence stem of the *Arabidopsis* transformants. Xyl, xylem; IF, interfascicular fibers; Pi, pith parenchyma cells; Ep, epidermis and cortex; Phl, phloem. Bar = 50 μm. (**B**) Total lignin content of the primary inflorescence and rosette leaves of *Arabidopsis* transformant lines determined by the acetyl bromide assay. A standard *t*-test analysis was conducted to identify statistically significant differences between the inflorescence stem and rosette leaf samples of each transformant line compared to Col-0 plants with the asterisks above a histogram column representing a *p*-value of * <0.05, ** <0.005, and *** <0.001.

Next, the anatomical differences between wild-type *Arabidopsis* and the *AtMIR397* and *SvMIR397* transformants were investigated via toluidine blue dye staining of cross-sections of the primary inflorescence stem (Figure 2A). The cross-sections of the primary inflorescence of transformants *AtMIR397* L2 and *SvMIR397* L1 exhibited anatomical features similar to those of the Col-0 primary inflorescence. In contrast, the primary inflorescence cross-sections of transformants *AtMIR397* L12 and L15 (the Group—2 and Group—3 representatives, respectively) revealed that these two transformants modified to overexpress the *Arabidopsis PRE-MIR397B* precursor sequence had developed inflorescence stems with a reduced diameter (Figure 2A). The reduced primary inflorescence stem diameter in these two transformants was believed to be the result of deformed and/or collapsed xylem, interfascicular fibers, and pith parenchyma cells. Highly similar developmental defects were observed in the Group—2 and Group—3 representative plants, *SvMIR397* L6 and L8, respectively, which had been molecularly modified to overexpress the *S. viridis PRE-MIR397B* precursor transcript (Figure 2A).

The anatomical defects displayed by the primary inflorescence stems of transformants *AtMIR397* L12, *AtMIR397* L15, *SvMIR397* L6, and *SvMIR397* L8 led us to next use phloroglucinol-HCl staining to investigate the degree of variation in the lignin content of these plant lines for comparison to wild-type *Arabidopsis*. Phloroglucinol and HCl are known to react with lignin to develop a pink-colored product, which can be used to visualize the lignified regions of plant cell walls. Figure 2A clearly shows that in all seven plant lines analyzed, including Col-0 plants, lignin deposition was localized to the xylem and interfascicular fibers, while no phloroglucinol-HCl staining was observed in the epidermis, cortex, phloem, or pith parenchyma cells, indicating that no lignin deposition occurred in these primary inflorescence structures. When compared to the cross-sections of the primary inflorescence of Col-0 plants, the level of lignification (as indicated by the pink-colored staining) varied dramatically across the three *AtMIR397* and *SvMIR397* phenotypic groups analyzed (Figure 2A). Further, a reduction to the total lignin content was readily apparent in the cross-section of the primary inflorescence stem sampled from each phenotypic group representative. More specifically, the mild phenotype group representatives, *AtMIR397* L2 and *SvMIR397* L1, only exhibited a slight reduction in the level of phloroglucinol-HCl staining in both the xylem and interfascicular fibers. It was interesting to observe that in the Group—2 representatives, *AtMIR397* L12 and *SvMIR397* L6, in spite of these two transformant lines displaying a phenotype of moderate severity, the degree of phloroglucinol-HCl staining of cross-sections of these two plant lines did not appear to differ from those obtained for the mild phenotype group representatives, transformants *AtMIR397* L2 and *SvMIR397* L1 (Figure 2A). Figure 2A further readily reveals the marked reduction in lignin deposition in the primary inflorescence stems of the two severe phenotype group (Group—3) representative plant lines *AtMIR397* L15 and *SvMIR397* L8.

In order to quantify the visualized change in lignin deposition in the primary inflorescence stem of *AtMIR397* and *SvMIR397* transformants for comparison to wild-type *Arabidopsis*, the total lignin content of the stems of Col-0, *AtMIR397*, and *SvMIR397* plants was determined via use of an acetyl bromide assay [31]. This quantitative approach clearly revealed that the lignin content of the inflorescence stem was significantly reduced in each of the six assessed plant lines selected to represent the three phenotypic groupings of the *AtMIR397* and *SvMIR397* transformant populations compared to that of wild-type *Arabidopsis* plants (Figure 2B). Further, and as expected, the acetyl bromide assay revealed that the two severe phenotype group representatives, *AtMIR397* L15 and *SvMIR397* L8, had the greatest degree of reduction to total lignin content (Figure 2B). More specifically, the total lignin content of the *AtMIR397* L15 and *SvMIR397* L8 primary inflorescence stem was significantly reduced by 47.9 and 49.2%, respectively, compared to the lignin content of the primary inflorescence stem of Col-0 plants.

Considering that rosette development also appeared altered by the overexpression of either the *Arabidopsis* or *S. viridis PRE-MIR397B* precursor transcript, especially the rosettes of transformants *AtMIR397* L12, *AtMIR397* L15, *SvMIR397* L5, and *SvMIR397* L8, the acetyl bromide assay was additionally used to determine the total lignin content of the rosette leaves of the six transformant lines selected for analysis in this study. As documented for the primary inflorescence stem of these transformant lines, total lignin was determined to be significantly reduced in the rosette leaves of all six transformant lines compared with that of Col-0 rosette leaves (Figure 2B). Although not to the same extent as documented for the inflorescence stem tissues, the greatest degree of reduction to total lignin content of the rosette leaves was determined for the severe phenotypic grouping representatives *AtMIR397* L15 and *SvMIR397* L8, with the rosette leaf lignin content of these two transformant lines reduced by 40.5 and 42.4%, respectively (Figure 2B).

### 2.3. Molecular Assessment of Representative AtMIR397 and SvMIR397 Transformant Lines

**Figure 3:**
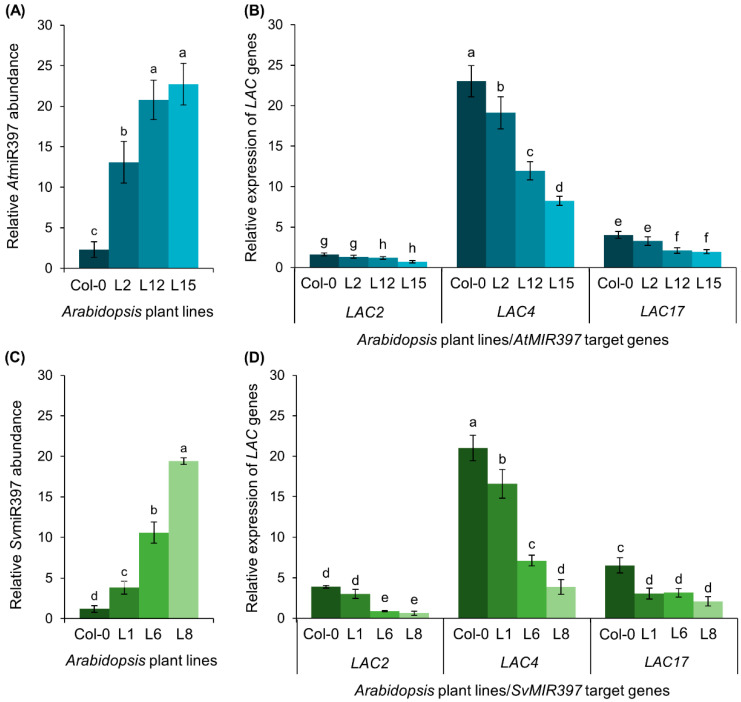
RT-qPCR quantification of miR397 sRNA abundance and *LACCASE* (*LAC*) target gene expression in the inflorescence stems of *AtMIR397* and *SvMIR397* transformant lines. (**A**) Abundance of the miR397 sRNA in *AtMIR397* transformants. (**B**) *LAC* target gene expression in *AtMIR397* transformants. (**C**) Abundance of the miR397 sRNA in *SvMIR397* transformants. (**D**) *LAC* target gene expression in *SvMIR397* transformants. Statistical data was analyzed using one-way ANOVA and Tukey’s post hoc tests. The statistically significant differences are indicated by a different letter (*p-*value < 0.05) above each of the different colored columns of each histogram.

A standard RT-qPCR approach was used to assess miR397 sRNA abundance, and the expression level of its targeted *LAC* genes, *LAC2*, *LAC4*, and *LAC17*, in the representative *AtMIR397* and *SvMIR397* transformant lines (Figure 3). Compared to its level in the inflorescence stem of Col-0 plants, RT-qPCR revealed the miR397 sRNA to be more abundant in the corresponding tissue of each of the six transformant lines assessed (Figure 3A,C). More specifically, miR397 abundance was determined to be elevated by 5.6-, 8.9-, and 9.7-fold in the inflorescence stem of *AtMIR397* transformants L2, L12, and L15, respectively (Figure 3A). A similar trend in miR397 sRNA abundance was documented for the three *SvMIR397* transformant lines assessed. That is, the miR397 sRNA was elevated in its abundance by 3.2-, 8.9-, and 16.3-fold in the *SvMIR397* transformant lines, L1, L6, and L8, respectively (Figure 3C). The increasing elevation in miR397 sRNA abundance in proportion to the severity of the developmental phenotype displayed by each of the analyzed *AtMIR397* and *SvMIR397* transformant lines strongly suggested that the observed developmental defects were the direct result of elevated miR397 sRNA accumulation in each transformant line analyzed in this study.

The miR397 sRNA has been shown to be a transcriptional regulator of *LAC* gene expression as part of the lignin polymerization process in *Arabidopsis* [26]. In addition, both *Arabidopsis* and *S. viridis* possess a highly similar mature miR397 sRNA sequence, which only differs by 2-nt across the 21-nt of the mature miR397 sRNA sequence (Figure S1). For these reasons, the ability of the *SvMIR397* sRNA to regulate the expression of the *Arabidopsis LAC2, LAC4*, and *LAC17* transcripts was next investigated via RT-qPCR in the inflorescence stem of *AtMIR397* and *SvMIR397* transformant lines for its comparison to unmodified wild-type *Arabidopsis* plants (Figure 3). The RT-qPCR-generated data revealed that *LAC2*, *LAC4*, and *LAC17* expression was reduced inversely proportional to the degree of increase in the accumulation of the miR397 sRNA in *AtMIR397* and *SvMIR397* transformants (Figure 3B,D). That is, the expression level of the *LAC* target genes was reduced to the greatest degree in the transformant lines that accumulated the highest level of the miR397 sRNA. More specifically, in the Group—1 representative, transformant *AtMIR397* L2, the expression level of each *LAC* target gene was mildly reduced by approximately 1.2-fold (Figure 3B) in response to the 5.6-fold elevation to miR397 abundance (Figure 3A). In the Group—2 representative *AtMIR397* L12, where miR397 abundance was determined to be elevated by 8.9-fold (Figure 3A), *LAC2* expression was reduced by 1.4-fold and the expression level of both *LAC4* and *LAC17* was reduced by 1.9-fold (Figure 3B). The highest degree of miR397 over-accumulation (enhanced by 9.7-fold) was unsurprisingly revealed by RT-qPCR to occur in transformant line *AtMIR397* L15, the Group—3 representative (Figure 3A). Accordingly, *LAC* target gene expression was subsequently determined to be most highly repressed in this transformant line, with the abundance of the *LAC2*, *LAC4*, and *LAC17* transcripts reduced by 2.3-, 2.8-, and 2.1-fold, respectively (Figure 3B), compared to the expression of each of these miR397 *LAC* target genes in the inflorescence stem of Col-0 plants. 

A similar miR397 accumulation, and *LAC* target gene expression profile, was generated by RT-qPCR for the three assessed representative lines of the *SvMIR397* transformant population (Figure 3C,D). In transformant *SvMIR397* L1, RT-qPCR revealed that the abundance of the introduced *S. viridis* miR397 sRNA was enhanced by 3.2-fold (Figure 3C), and in response to this enhancement, *LAC2*, *LAC4*, and *LAC17* expression was repressed by 1.3-, 1.3-, and 2.1-fold, respectively (Figure 3D). Transformant line *SvMIR397* L6, the Group—2 representative, was determined to have 8.9-fold higher miR397 abundance than that of wild-type *Arabidopsis* plants at the same stage of development (Figure 3C). In accordance with this elevated miR397 abundance, *LAC2*, *LAC4*, and *LAC17* expression was subsequently revealed by RT-qPCR to be reduced by 4.4-, 3.0-, and 2.1-fold, respectively (Figure 3D) in the *SvMIR397* L6 transformant. In the severe phenotype group (Group—3) representative, the *SvMIR397* L8 transformant, miR397 levels were elevated by 16.3-fold and *LAC2*, *LAC4*, and *LAC17* expression was significantly reduced by 6.3-, 5.4-, and 3.2-fold, respectively, compared to non-modified Col-0 plants (Figure 3C,D). When taken together, the expression analyses presented in Figure 3 clearly show that the (1) miR397 sRNA regulates the expression of its targeted genes via the canonical mechanism of miRNA-directed RNA silencing in *Arabidopsis* (miRNA-directed target transcript cleavage), and (2) high level of sequence similarity of the introduced miR397 sRNA from *S. viridis*, to that of the endogenous *Arabidopsis* miR397, allowed the *SvMIR397* sRNA to efficiently regulate the expression of the *Arabidopsis* miR397 target genes, *LAC2*, *LAC4*, and *LAC17*, at the posttranscriptional level.

### 2.4. Phenotypic and Physiological Assessment of AtMIR397 and SvMIR397 Transformant Lines Exposed to Salt Stress

**Figure 4:**
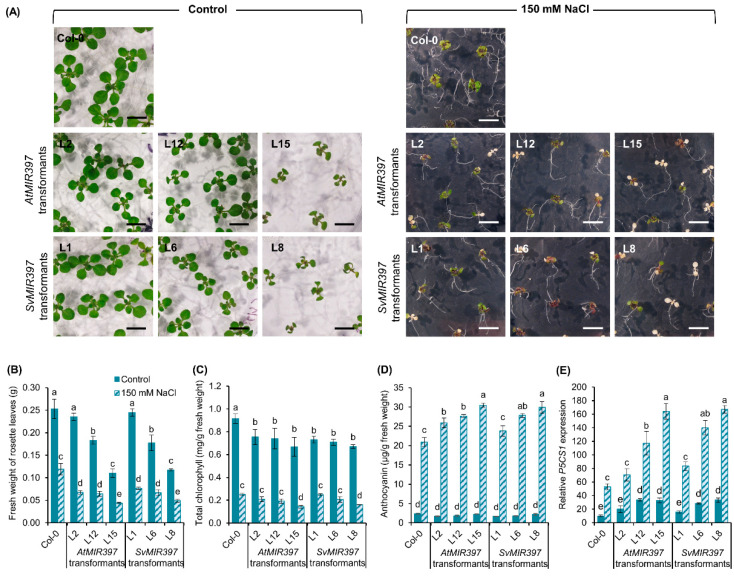
Phenotypic and physiological assessment of 15-day-old *AtMIR397* and *SvMIR397* transformant lines post their exposure to a 7-day salt stress treatment regime. (**A**) Phenotypes displayed by 15-day-old control (left hand side panels) and salt-stressed (right hand side panels) wild-type *Arabidopsis* plants, and *AtMIR397* and *SvMIR397* transformant lines. Bar = 1.0 cm. Assessment of the phenotypic and physiological parameters, (**B**) rosette fresh weight (g), (**C**) total chlorophyll (mg/g fresh weight), and (**D**) anthocyanin (µg/g fresh weight). (**E**) Evaluation of the expression of the oxidative stress response gene, *P5CS1*. Statistical data were analyzed using one-way ANOVA and Tukey’s post hoc tests. The statistically significant differences are indicated by a different letter (*p*-value < 0.05) above each column of each histogram.

To investigate the effect of salt stress on *Arabidopsis* plants molecularly manipulated to over-accumulate the miR397 sRNA, we transferred eight-day-old seedlings from each representative of the three phenotypic groupings of the *AtMIR397* (L2, L12, and L15) and *SvMIR397* (L1, L6, and L8) transformant populations to either a fresh plate of standard *Arabidopsis* growth media or to a plate that contained *Arabidopsis* growth medium that had been supplemented with 150 mM sodium chloride (NaCl) for the salt stress treatment regime. Post-seedling transfer to either standard (control) or salt stressed medium, we cultivated *Arabidopsis* plants for an additional 7-day period. Figure 4A clearly shows that in comparison to control-grown Col-0, *AtMIR397*, and *SvMIR397* seedlings, the development of shoot architecture was severely inhibited in all seedlings post the 7-day stress treatment period. Specifically, the total rosette area of all salt-stressed seedlings was noticeably reduced compared to that of the non-stressed, control-grown counterpart of each plant line. It was therefore unsurprising that quantification of the fresh weight of salt-stressed Col-0 seedlings revealed a 52.9% reduction to this phenotypic parameter in comparison to control-grown Col-0 plants (Figure 4B). The ability of a 7-day 150 mM NaCl stress growth regime to inhibit shoot growth was determined to be more severe for the *AtMIR397* and *SvMIR397* transformant populations. Specifically, compared to their control-grown, non-stressed counterparts, the fresh weight of the rosette was significantly reduced by 71.3, 64.8, and 60.3% for transformant lines *AtMIR397* L2, L12, and L15, respectively (Figure 4B). Similarly, for the three assessed *SvMIR397* representatives, transformants L1, L6, and L8, the fresh weight of the rosette was determined to be reduced by 68.7, 62.3, and 56.2%, respectively, after the 7-day 150 mM NaCl stress treatment (Figure 4B).

The applied 7-day 150 mM NaCl stress regime was also determined to greatly reduce the total chlorophyll content of each assessed transformant line. The reduction in total chlorophyll content was readily visible by the presence of photobleached rosette leaves, a phenotypic response particularly apparent for the six *AtMIR397* and *SvMIR397* transformants (Figure 4A), compared to salt-stressed Col-0 plants. Furthermore, amongst the six assessed transformant lines, the two Group—3 representatives of each transformant population, namely, the *AtMIR397* L15 and *SvMIR397* L8 transformants, readily displayed a higher number of completely photobleached rosette leaves compared to either the Group—1 or Group—2 representatives, or to wild-type *Arabidopsis* plants (Figure 4A). In order to quantify the visually obvious reduction in total chlorophyll, spectrophotometry was utilized, with this analysis revealing that in comparison to their control grown counterparts, the total chlorophyll content of Col-0 plants was reduced by 72.0% (Figure 4C). In the transformant lines, the total chlorophyll content of *AtMIR397* transformants L2, L12, and L15 was reduced by 72.2, 74.0, and 78.5%, respectively, while the total chlorophyll content of the *SvMIR397* transformants L1, L6, and L8 was reduced by 65.9, 70.9, and 76.2%, respectively (Figure 4C).

In direct contrast to the decrease in total chlorophyll content determined for each *Arabidopsis* plant line grown in the presence of 150 mM NaCl, a marked increase in the antioxidant anthocyanin was present in the rosette tissue of the same salt-stressed plants when compared to their respective control-grown counterpart (Figure 4D). For example, 15-day-old control-grown Col-0 seedlings were determined to have an anthocyanin content of 2.4 μg per gram of fresh weight; however, post the 7-day cultivation period in the presence of 150 mM NaCl, the anthocyanin content of salt-stressed Col-0 seedlings increased to 21.0 μg per gram of fresh weight, an 8.9-fold elevation in the abundance of this antioxidant pigment (Figure 4D). In comparison to the wild-type findings, and with respect to the control-grown counterpart of the three *AtMIR397* transformant lines analyzed, the accumulation of anthocyanin was further promoted by 15.6-fold in transformants *AtMIR397* L2 and L12, and by a slightly lesser degree, 13.6-fold, in transformant *AtMIR397* L15 (Figure 4D). A highly similar accumulation profile was obtained for the three representative lines molecularly manipulated to over-accumulate the *S. viridis* miR397 sRNA. Namely, compared to the control-grown counterpart of each transformant line, anthocyanin content was elevated by 14.4-, 15.4-, and 13.8-fold in transformants *SvMIR397* L1, L6, and L8, respectively, after their cultivation for a 7-day period in the salt stress growth environment (Figure 4D).

The reduced chlorophyll and elevated anthocyanin content of each plant line after its exposure to the applied salt stress treatment indicated that at the physiological level, each assessed plant line was indeed stressed. We therefore next used a standard RT-qPCR approach to confirm that each plant line was also experiencing “stress” at the molecular level. Initially, RT-qPCR revealed that the expression of the *Arabidopsis* stress marker gene *P5CS1* was elevated in each of the six analyzed transformant lines, compared to its expression in Col-0 plants, when all seven plant lines were cultivated for 15 days in control growth conditions (Figure 4E). Subsequently, RT-qPCR further showed that in spite of *P5CS1* expression already being higher in control-grown transformants than it is in the Col-0 control, the expression of *P5CS1* was promoted to a similar degree in all analyzed plant lines after the application of salt stress. Specifically, compared to the control grown counterpart of each assessed plant line, *P5CS1* expression was elevated by 5.5-, 3.5-, 3.5-, 5.0-, 5.3-, 4.9-, and 5.0-fold in salt-stressed Col-0, *AtMIR397* L2, *AtMIR397* L12, *AtMIR397* L15, *SvMIR397* L1, *SvMIR397* L6, and *SvMIR397* L8 plants, respectively (Figure 4E).

### 2.5. Molecular Assessment of AtMIR397 and SvMIR397 Transformant Lines Exposed to Salt Stress

**Figure 5:**
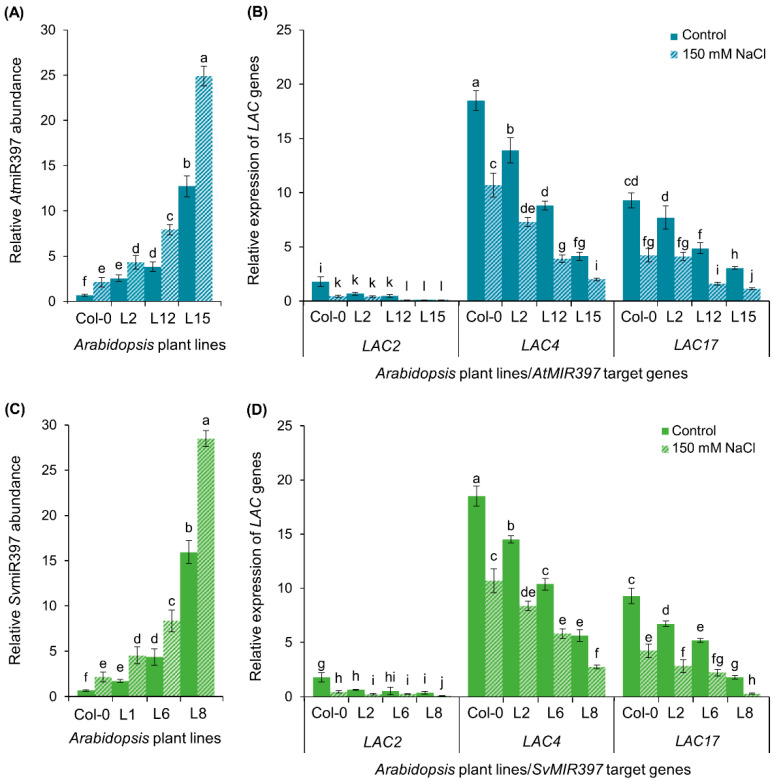
Quantification of miR397 sRNA abundance and *LACCASE* target gene expression in 15-day-old *AtMIR397* and *SvMIR397* transformant lines cultivated under standard growth conditions or after their exposure to salt stress. (**A**) Quantification of miR397 abundance in control grown and salt-stressed *AtMIR397* transformants. (**B**) RT-qPCR analysis of *LAC* target gene expression in control grown and salt-stressed *AtMIR397* transformants. (**C**) RT-qPCR assessment of the abundance of the miR397 sRNA in control and salt-stressed *SvMIR397* transformants. (**D**) Expression of the three miR397-targeted *LAC* genes in control or salt-stressed 15-day-old *SvMIR397* transformants. Statistical data were analyzed using one-way ANOVA and Tukey’s post hoc tests. The statistically significant differences (*p*-value < 0.05) are indicated by a different letter above each column of each histogram.

RT-qPCR analyses were conducted to evaluate the accumulation trend of the miR397 sRNA and to construct expression profiles for its three *LAC* target genes in 15-day-old wild-type *Arabidopsis* plants and the *AtMIR397* and *SvMIR397* transformant lines cultivated under standard growth conditions or after their exposure to the 7-day salt stress treatment regime. Determination of miR397 abundance revealed that the miR397 sRNA returned a highly similar accumulation pattern across the three *AtMIR397* and *SvMIR397* representative transformant lines analyzed under standard growth conditions or when exposed to the salt stress treatment. More specifically, compared to 15-day-old Col-0 control plants, miR397 sRNA abundance was elevated by 3.8-, 5.7-, and 18.9-fold in control-grown *AtMIR397* transformant lines L2, L12, and L15, respectively (Figure 5A). A similar trend of incrementally increased miR397 sRNA abundance was observed across the three phenotype group representatives assessed for the *SvMIR397* transformant population with miR397 levels elevated by 2.6-, 6.4-, and 23.6-fold in control-grown *SvMIR397* transformant lines L1, L6, and L8, respectively (Figure 5C). Further enhancement to miR397 abundance in all six transformant lines analyzed in this study after their exposure to the 7-day 150 mM NaCl stress treatment was unsurprising considering that RT-qPCR revealed miR397 sRNA levels to be elevated by 3.2-fold in salt-stressed Col-0 plants compared to Col-0 control plants (Figure 5A,C).

After determination of the miR397 accumulation trend in control and salt-stressed *AtMIR397* and *SvMIR397* transformants, we next used RT-qPCR to quantify *LAC2*, *LAC4*, and *LAC17* target gene expression in these plant lines cultivated under standard growth conditions or after their exposure to salt stress. This approach revealed a highly reciprocal relationship between the miR397 sRNA and the expression level of its three *LAC* target genes, regardless of the growth regime. More specifically, miR397 accumulated to its lowest level in non-modified, control-grown wild-type *Arabidopsis* (Figure 5A,C), and accordingly, *LAC2*, *LAC4*, and *LAC17* were expressed to their highest degree in Col-0 control plants (Figure 5B,D). Furthermore, and taking the *SvMIR397* transformant population as an example, RT-qPCR subsequently revealed the miR397 sRNA to accumulate to its highest level in the Group—3 representative *SvMIR397* L8 (upregulated by 23.6-fold), and for the *LAC17* target gene to be expressed at its lowest level (downregulated by 5.6-fold) in this transformant line after its exposure to salt stress (Figure 5B,D). A similar incremental enhancement to miR397 abundance and repression of target gene expression was documented for the *LAC2* and *LAC4* target genes in control and salt-stressed transformants *SvMIR397* L1 and *SvMIR397* L6, and for the three *AtMIR397* transformant lines, across the two growth regimes, also analyzed as part of this assessment. 

## 3. Discussion

### 3.1. MicroRNA397 is the Posttranslational Regulator of the Expression of LACCASE Genes Involved in Lignin Polymerization

LACCASE glycoproteins belong to the multi-copper oxidase protein superfamily and are involved in a wide range of physiological processes in plants [32,33]. Since the 1960s, numerous LAC proteins have been identified and annotated, and several of these have been found to play a critical role in catalyzing lignin polymerization in *Arabidopsis* [5,25,33], rice [34], *Brachypodium distachyon* [35], sorghum [36], and poplar (*Populus trichocarpa*) [27]. More recently, the highly conserved regulatory sRNA, miR397, has been demonstrated to regulate the expression of several *LAC* gene family members at the posttranscriptional level in *Arabidopsis* [26,37] and poplar [27]. For example, the over-accumulation of the miR397 sRNA has been reported to significantly downregulate the expression of *LAC* gene targets in molecularly modified plants, leading to reduced total lignin content (reduced by ≈47%), and the disruption of vascular structures in the resulting transformant lines [26,27]. The identification of the miR397/*LAC* expression module has opened a new avenue to further our current understanding of the mechanisms at the posttranscriptional level that regulate lignin polymerization in plant cell walls. However, to date, few studies have focused on the posttranscriptional regulation of *LAC* gene expression in C_4_ plant species, including *S. viridis*, a recently established model system for the continued genetic characterization of C_4_ crops and bioenergy feedstocks [38,39,40]. In this study, via the use of *Arabidopsis* as a heterologous system, we provide strong phenotypic and molecular evidence that the *S. viridis* miR397 sRNA is capable of functioning as a posttranscriptional regulator of the expression of the *Arabidopsis LAC* gene family members *LAC2*, *LAC4*, and *LAC17*.

We next used a standard bioinformatic approach to identify a putative miR397 precursor transcript, that we termed *PRE-MIR397B* due to its high degree of homology to the *Arabidopsis PRE-MIR397B* precursor transcript, and subsequently determined that the identified transcript was encoded by a locus harbored by *S. viridis* chromosome 1 (Figure S1). The mature *S. viridis* miR397 sRNA was subsequently determined to be a highly conserved sequence of 22-nt in length, due to this sRNA possessing one additional nucleotide at its 3′ terminus, compared to 21-nt mature miR397 sRNA of *Arabidopsis*. In addition to carrying an additional nucleotide at its 3′ end, the alignment of the *S. viridis* and *Arabidopsis* miR397 sRNA sequences revealed that *SvMIR397* harbored a single nucleotide mismatch to the *At*miR397 sRNA at nt position 13 (Figure S1). Alignment of the *SvMIR397* sRNA to the *Arabidopsis LAC2, LAC4*, and *LAC17* transcripts, as well as to the *S. viridis* homologs of the *Arabidopsis LAC4* and *LAC17* transcripts, also revealed a high degree of complementarity; a finding that suggested that the *SvMIR397* sRNA could potentially function as a posttranscriptional regulator of *LAC* gene expression in *Arabidopsis* (Figure S1). 

Substantial evidence for the role of the *SvMIR397* sRNA in the posttranscriptional regulation of *LAC* gene expression in *Arabidopsis* was provided by the RT-qPCR analyses performed on the *SvMIR397* transformant lines and their comparison to both the *AtMIR397* transformants, as well as to wild-type *Arabidopsis*. Specifically, RT-qPCR revealed a significant over-accumulation of the *SvMIR397* sRNA in *SvMIR397* transformants molecularly modified to overexpress the *PRE-MIR397B* precursor transcript from *S. viridis*. On the basis of the RT-qPCR findings, we divided *SvMIR397* transformants into three phenotypic groupings, the Group—1 (wild-type-like), Group—2 (semi-dwarfism), and Group—3 (full dwarfism) phenotypic groupings (Figure 1), with the mildest degree of miR397 over-accumulation detected in the Group—1 representative, transformant *SvMIR397* L1, and the Group—3 representative, *SvMIR397* L8, determined to over-accumulate the introduced sRNA to the highest level in its primary inflorescence stem (Figure 3). The RT-qPCR analyses further revealed a very tight reciprocal relationship between *SvMIR397* abundance and *Arabidopsis LAC* target gene expression, that is, the greater the enhancement to *SvMIR397* abundance, the more significant the degree of *LAC2*, *LAC4*, and *LAC17* target gene expression repression (Figure 3). Together, the bioinformatic and molecular analyses of the *Arabidopsis* transformant lines molecularly modified to over-accumulate the miR397 sRNA from *S. viridis* strongly inferred that in *S. viridis*, miR397 plays a central regulatory role in the lignin biosynthesis pathway.

The molecular profiling of the miR397/*LAC* expression module across the representative *AtMIR397* and *SvMIR397* transformants, together with the subsequent comparison of this transformant data to that obtained from non-modified wild-type *Arabidopsis*, strongly suggested that the greatly elevated abundance of the miR397 sRNA, in parallel with the documented significant repression of *LAC* target gene expression, was likely responsible for the developmental phenotypes displayed by the miR397 over-accumulation lines. The visual inspection of lignin deposition via histochemical staining and the quantification of total lignin content via acetyl bromide assay analysis provided further supportive evidence of the regulatory role of the introduced *SvMIR397* sRNA in controlling *LAC* target gene expression, and therefore LAC-mediated lignin biosynthesis, in the generated *Arabidopsis* transformants (Figure 2). More specifically, the acetyl bromide approach used in this study to quantify total lignin readily revealed that the over-accumulation of the *SvMIR397* sRNA resulted in a reduction to the total lignin content in all transformant lines assessed, with total lignin content demonstrated to be reduced by as much as 49.2 and 42.4% in the primary inflorescence and rosette leaves of *SvMIR397* transformants, respectively (Figure 2B). Furthermore, the reduction to total lignin content was believed to be the cause of the irregular morphology of xylem, interfascicular fibers, and pith cells in the assessed *Arabidopsis* transformant lines, especially those lines displaying moderate (Group—2, *AtMIR397* L12 and *SvMIR397* L6) and severe (Group—3, *AtMIR397* L15 and *SvMIR397* L8) developmental phenotypes (Figure 2A). It is also interesting to note that the over-accumulation of either the *Arabidopsis* or *S. viridis* miR397 sRNA resulted in an increase the size of seeds produced by the moderate and severe phenotype expressing transformant lines *AtMIR397* L12, *AtMIR397* L15, *SvMIR397* L6, and *SvMIR397* L8 (Figure 1B,G). Similar observations have been reported previously for *Arabidopsis* [26] and rice [41] plants molecularly modified to overexpress the *PRE-MIR397B* precursor transcript. The enlargement of seed size has been suggested to likely result from the enhancement of the concentration of the brassinosteroid signal, a phytohormone essential for plant growth, development, and environmental adaptations, and which forms a process coincidently activated by the increased abundance of the miR397 sRNA in the inflorescence stem and floral tissues of these two model plant species [26,41].

### 3.2. The AtMIR397 and SvMIR397 Transformant Lines Molecularly Modified to Over-Accumulate the MicroRNA397 Small RNA Are More Sensitive to Salt Stress

The phenotypic and physiological analyses reported in Figure 4 readily reveal the detrimental effects of salt stress on the growth and development of the *AtMIR397* and *SvMIR397* transformant lines. Under salt stress treatment, all six transformant lines assessed, experienced a dramatic reduction in plant growth and biomass, as indicated by the reduced size and fresh weight of their rosettes (Figure 4). More specifically, the fresh weight of *AtMIR397* and *SvMIR39* transformant lines was reduced by up to ≈71 and ≈69%, respectively, by the 7-day cultivation period on *Arabidopsis* growth media which had been supplemented with 150 mM NaCl (Figure 4B). The restriction to the growth and development of salt-stressed *AtMIR397* and *SvMIR397* transformants could be, in part, attributed to the inhibition of cell division [42,43], a commonly reported physiological consequence of salt stress believed to be the result of excessive damage to the nuclear DNA in the meristematic cells of many plant species, including *Arabidopsis* [44], S*olanum lycopersicum* (tomato) [45], *Hordeum vulgare* (barley) [46], and *Secale cereale* (rye) [47]. In addition, when subjected to a high level of salt stress, plants also experience osmotic stress, another factor known to impede plant growth. Osmotic stress restricts the uptake and transportation of water into plants, thereby reducing the water potential and essential turgor pressure of plant cells, and as a consequence, plant cell elongation and expansion are inhibited [48,49].

Figure 4 also clearly shows the significant reduction in total chlorophyll content recorded for each assessed *AtMIR397* and *SvMIR397* transformant following the exposure of these plant lines to the salt stress treatment regime (Figure 4C); a finding that is in agreement with previous studies in *Arabidopsis* [12,19], sorghum [9], and *Phaseolus vulgaris* (common bean) [42]. The significant reduction, to complete dysfunction, of the photosynthetic capacity of salt-stressed *AtMIR397* and *SvMIR397* seedlings was clearly evident by the presence of partially and fully photobleached rosette leaves, with the severe phenotypic group (Group—3) representatives, transformants *AtMIR397* L15 and *SvMIR397* L8, displaying a considerably higher number of completely damaged rosette leaves compared to the representative plants of the two other phenotypic groupings (Group—1 or Group—2 representatives) also assessed, or to salt-stressed Col-0 seedlings (Figure 4A). The decrease in the total chlorophyll content in the salt-stressed transformants was likely due to the (1) inhibition of chlorophyll biosynthesis, together with (2) rapid degradation of the existing chlorophyll, a commonly reported consequence of oxidative stress resulting from the over-accumulation of ROS [42,50]. In response to salt stress, plants have developed a series of defense mechanisms to alleviate ROS toxicity and to protect cells from potential oxidative damage [42]. To this end, anthocyanin is a natural antioxidant that accumulates to very high levels in plant cells under salt stress-induced oxidative damage to maintain in vivo ROS homeostasis and to attempt to prevent the degradation of chlorophyll [30,51]. As expected, on the basis of the findings of the fresh weight and total chlorophyll content analyses, anthocyanin significantly increased in abundance in the aerial tissues of all assessed plant lines, with the greatest degree of anthocyanin accumulation enhancement recorded for the Group—3 representatives *AtMIR397* L15 and *SvMIR397* L8 (Figure 4D).

Having clearly demonstrated the ability of the introduced *SvMIR397* sRNA to act as a posttranscriptional regulator of *Arabidopsis LAC2*, *LAC4*, and *LAC17* gene expression, we reassessed the abundance of the miR397 sRNA and the expression of its three *LAC* target genes by RT-qPCR to determine if the enhanced phenotypic and physiological sensitivity of *AtMIR397* and *SvMIR397* transformant lines to the applied salt stress treatment regime was the result of further alteration to the miR397/*LAC* regulatory module in these plant lines. This analysis clearly revealed that miR397 abundance was elevated, and that the expression of its three *LAC* target genes was repressed, in all seven plant lines analyzed in this study after their exposure to the salt stress treatment regime (Figure 5). This result is consistent with our previous studies where we reported on the molecular profiling of the miRNA landscape of salt-stressed wild-type *Arabidopsis* and *S. viridis* seedlings [19,20]. In these two studies, the abundance of the miR397 sRNA was determined to be significantly upregulated in both model plant species after their exposure to salt stress [19,20]. The increase in miR397 abundance in response to salt stress is believed to act as one of the defense mechanisms that a plant uses to attempt to combat the negative impact of salt stress with elevated miR397 levels directing the repression of *LAC* target gene expression, leading to reduced LAC enzyme activity, and therefore, reduced lignification of plant cell walls to enhance their plasticity [48,52]. Reduction in the deposition of lignin in the secondary cell wall could lead to the generation of a network of relatively thinner, more flexible, and therefore more water permeable cell walls, characteristics that would more readily enable osmotic adjustment to maintain cell turgor pressure to allow for the maintenance of cell elongation and/or expansion during periods of salt stress-induced osmotic stress [48,52].

### 3.3. The Use of the Arabidopsis thaliana Heterologous System for Plant Molecular Biology Studies

A result of particular interest observed in this study stemmed from the comparison of the molecular profiling of the moderate phenotypic group (Group—2) representatives of the *AtMIR397* and *SvMIR397* transformant populations, namely, plant lines *AtMIR397* L12 and *SvMIR397* L6 (Figure 3). In both transformant lines, the miR397 sRNA was determined to be elevated in its abundance by 8.9-fold (Figure 3A,C). In transformant line *AtMIR397* L12, the 8.9-fold elevation in miR397 abundance reduced *LAC2*, *LAC4*, and *LAC17* target gene expression by 1.4-, 1.9-, and 1.9-fold, respectively (Figure 3A,B). However, although miR397 sRNA abundance was enhanced by the same degree in transformant line *SvMIR397* L6, *LAC2*, *LAC4*, and *LAC17* target gene expression was reduced by a greater extent, down by 4.4-, 3.0-, and 2.1-fold, respectively (Figure 3C,D). Alignment of the *S. viridis* miR397 sRNA sequence to those of the *Arabidopsis LAC2*, *LAC4*, and *LAC17* target transcripts revealed that the *S. viridis* miR397 sRNA possesses (1) a higher degree of complementarity to the targeted *Arabidopsis LAC* transcripts, and (2) mismatched base-pairings to the *LAC* target gene transcripts in more favorable positions (toward the end of the 3′ half of the targeting sRNA) than that of the endogenous *Arabidopsis* miR397 sRNA (Figure S1C,D), characteristics that would be expected to direct tighter regulation of target gene expression by the sRNA. It is also interesting to note from this alignment analysis that the *S. viridis* miR397 has a greater degree of complementarity to the *Arabidopsis LAC* target gene transcripts than this sRNA does to its own endogenous target gene transcripts, including the *LAC4-1*, *LAC4-2*, *LAC4-3*, *LAC4-4*, *LAC17-1*, *LAC17-2*, and *LAC17-3* target transcripts that we bioinformatically identified from the currently available *S. viridis* transcriptomic resources (Figure S1E). The use of *Arabidopsis* as a heterologous system in this study did, however, readily allow for the desired demonstration that the miR397 sRNA of *S. viridis* is able to regulate the expression of the *Arabidopsis* miR397 genes *LAC2*, *LAC4*, and *LAC17* at the posttranscriptional level. Therefore, the *S. viridis* miR397 sRNA would be expected to perform a similar regulatory function at the posttranscriptional level to control *LAC* target gene expression in *S. viridis*. The recent demonstration [53,54,55,56] that *S. viridis* is amenable to molecular modification by *Agrobacterium tumefaciens*-mediated transformation has opened the door for further characterization of the miR397/*LAC* target gene relationship in the C_4_ monocotyledonous grasses. More specifically, it can be envisaged that the molecular manipulation of *S. viridis* would provide significant additional biological insight into miRNA-directed posttranscriptional regulation of the lignin biosynthesis pathway in the C_4_ grasses, a group of plants that includes agronomically important species such as maize, sorghum, and sugarcane (*Saccharum officinarum*); biological insight that cannot be obtained from the use of a C_3_ species such as *Arabidopsis* as an genetic model.

An additional finding presented in this study that warrants further investigation in the newly established genetic model, *S. viridis*, is the demonstration that the over-accumulation of the miR397 sRNA from either *Arabidopsis* or *S. viridis* resulted in the production of larger sized seeds in the moderate and severe phenotypic group representatives, including the transformant lines *AtMIR397* L12, *AtMIR397* L15, *SvMIR397* L6, and *SvMIR397* L8 (Figure 1B,G). However, in parallel to displaying the desirable phenotype of producing seeds of increased size, the *AtMIR397* and *SvMIR397* transformant lines additionally expressed the unwanted phenotype of semi- to full-dwarfism characterized by a reduction to their overall biomass stemming from smaller sized and abnormally shaped rosette leaves that were reduced in number, together with the formation of a shorter primary inflorescence stem that lacked branching and the inhibition of the formation of secondary inflorescences (Figure 1A,C–E). The generated *AtMIR397* and *SvMIR397* transformant lines were also demonstrated to be more sensitive to the imposed 7-day salt stress treatment regime (Figure 4). Previous research has shown that a reduced lignin content can be beneficial to a plant when exposed to salt stress with the increased flexibility of secondary cell walls allowing for greater cell wall water permeability, and therefore more ready adjustment of the osmotic potential of these cells in order for the cells to maintain turgor pressure. This in turn would allow for these cells to continue to elongate and expand during the period of salt stress exposure [48,52]. However, the demonstration that the *AtMIR397* and *SvMIR397* transformant lines were more sensitive to the imposed stress, than were unmodified wild-type *Arabidopsis* plants, strongly suggests that there is a threshold to the benefit of reduced lignin content in plant secondary cell walls and that the transformant lines generated in this study had greatly surpassed that threshold (Figure 2 and Figure 4). Therefore, in future research endeavors using a molecular approach in the newly established *S. viridis* genetic model, tissue-specific promoters could be used to drive the over-accumulation of the miR397 sRNA in select tissues of *S. viridis* to determine (1) in which tissues, and (2) to what degree, the over-accumulation of the miR397 sRNA allows for the molecularly modified *S. viridis* plants to display the desired phenotypic characteristics reported here in *Arabidopsis* miR397 over-accumulating lines while avoiding the expression of those unwanted developmental and stress response phenotypes also observed in these transformant lines. Determination of these parameters using the *S. viridis* model could prove to be of extreme benefit to future agriculture, considering the close relatedness of *S. viridis* to other C_4_ monocot grasses of agronomic importance, including maize, sorghum, and sugarcane.

### 3.4. Study Summary and Perspectives

In summary, here we clearly demonstrate that the *S. viridis* miR397 can act as a posttranscriptional regulator of the expression of the three known *Arabidopsis* miR397 *LAC* target genes when *Arabidopsis* is molecularly manipulated to over-accumulate the *S. viridis* miR397 sRNA. For both the *Arabidopsis* and *S. viridis* miR397 over-accumulating transformant populations, we document a clearly defined reciprocal relationship between the abundance of the regulating sRNA, miR397, to the expression level of its three *LAC* target genes. Furthermore, the degree of elevation to the accumulation of miR397, together with the extent to which *LAC2*, *LAC4*, and *LAC17* target gene expression was repressed, was demonstrated to strongly correlate to the severity of the developmental phenotype displayed by *AtMIR397* and *SvMIR397* transformant lines. The extent to which lignin deposition and total lignin content were reduced also tightly correlated with the assessed metrics of (1) elevated miR397 sRNA abundance, and (2) repressed *LAC* target gene expression in the *AtMIR397* and *SvMIR397* transformants lines. We have previously demonstrated that in *Arabidopsis* and *S. viridis*, the miR397 sRNA is responsive to salt stress [19,20], and here, each *Arabidopsis* plant line molecularly altered to have elevated miR397 abundance, and therefore repressed *LAC* target gene expression, appeared to be more sensitive to the applied salt stress treatment regime than were non-modified wild-type *Arabidopsis* plants, a sensitivity that is potentially the result of reduced lignin content in the secondary cell walls of the generated transformant lines leading to increased cell wall permeability and therefore promotion of osmotic stress penetration. In conclusion, the results presented here broaden our current understanding of the regulatory role that the miR397 sRNA directs at the posttranscriptional level to control *LAC* gene expression as part of the lignin biosynthesis pathway of *Arabidopsis* and *S. viridis*. Our findings further highlight the contribution of lignin as a mediating factor as part of the morphological response of a plant to adapt to salt stress. Furthermore, with the sequence and function of the miR397/*LAC* expression module shown to be highly conserved across a number of unrelated C_3_ and C_4_ plant species [26,37,41,57], the data presented in our study serves as an important starting resource for the future development of new agricultural plant lines that harbor molecular manipulations to the miR397/*LAC* expression module, or to other similar miRNA/target gene expression modules, that enable these plant lines to adapt to cultivation in saline soils, the prevalence of which is ever increasing globally.

## 4. Materials and Methods

### 4.1. Plant Material and Agrobacterium tumefaciens-Mediated Transformation of Arabidopsis thaliana

Wild-type seeds of *Arabidopsis thaliana* (ecotype; Columbia-0 (Col-0)) were surface sterilized with chlorine gas for 90 min in a sealed chamber. Surface-sterilized seeds were plated out onto half-strength Murashige and Skoog (MS) media plates and stratified for 48 hours (h) at 4 °C in the dark to ensure uniform germination and subsequent plant developmental progression. Post-stratification, the plates were transferred to a temperature-controlled growth cabinet (A1000 Growth Chamber, Conviron, Melbourne, Australia) and cultivated under a 16 h light (100–120 μmol m^−2^ s^−1^) and 8 h dark cycle with a day/night temperature of 22 °C/18 °C. Eight-day-old seedlings were subsequently transferred to soil (Seeds and Cuttings Mix, Debco, Tyabb, Australia), and maintained under the same growth conditions until Col-0 plants had progressed to the reproductive stage of development. The floral material was used for *Agrobacterium tumefaciens* (*Agrobacterium*)-mediated transformation.

*Agrobacterium*-mediated transformation of Col-0 plants was undertaken according to the protocol of [58]. The plant expression vectors used for *Arabidopsis* transformation were p*AtMIR397*-OE and p*SvMIR397*-OE. The p*AtMIR397*-OE and p*SvMIR397*-OE vectors were generated via placing an artificially synthesized (Integrated DNA Technologies, Sydney, Australia) *Arabidopsis* or *S. viridis PRE-MIR397B* encoding sequence behind the *Cauliflower mosaic virus* (*CaMV*) *35S* promoter in the pBART plant expression vector backbone (Figure S2). 

To screen for putative transformation events, the same germination procedure as outlined above was used, except that the half-strength MS media was supplemented with the selective agent, phosphinothricin (PPT), at a concentration of 10 mg/L. Successive generations of each obtained transformant line underwent the “selection” process until each plant line was confirmed to be homozygous for the introduced transgene. Eight-day-old seedlings of wild-type Col-0 plants and each assessed *AtMIR397* and *SvMIR397* transformant line were cultivated in soil pots under standard growth conditions until they reached the appropriate stage of development required to sample the plant material and conduct the reported experimental analyses.

For salt stress treatment, seeds of wild-type Col-0 plants and the selected *AtMIR397* and *SvMIR397* transformant lines were surface sterilized, stratified, and cultivated in sealed Petri dishes containing standard *Arabidopsis* growth medium (half strength MS media) in a temperature-controlled growth cabinet for 8 days. Following this 8-day cultivation period, an equal number of Col-0, *AtMIR397*, and *SvMIR397* seedlings were transferred to new plates that contained either (1) fresh standard *Arabidopsis* growth medium, or (2) fresh *Arabidopsis* growth medium supplemented with 150 millimolar (mM) sodium chloride (NaCl). Post seedling transfer, each plate was sealed with gas permeable tape and then returned to the temperature-controlled growth cabinet for an additional 7-day cultivation period using the lighting and temperature conditions as outlined above.

### 4.2. Phenotypic and Physiological Analyses of Arabidopsis Transformants

*Arabidopsis* transformant lines *AtMIR397* L2, *AtMIR397* L12, *AtMIR397* L15, *SvMIR397* L1, *SvMIR397* L6, and *SvMIR397* L8 were phenotypically assessed by measuring the length of the primary inflorescence stem and the primary root, the aerial tissue fresh weight, and the rosette area and seed size for comparison to unmodified Col-0 plants of the same age. The length of the primary root was measured for 15-day-old plants that had been grown for the entire 15-day period on standard *Arabidopsis* growth media, or which had been transferred to the salt stress growth regime at 8 days of age. For primary root length measurements, it is important to note here that after the transfer of 8-day-old seedlings to fresh control or salt stress media plates, we subsequently orientated the plates vertically to allow for this measurement to be accurately taken. Rosette leaf area of each transformant line was measured at reproduction stage (3 weeks post germination) using the Easy-Leaf-Area software [59]. Seed size (average of 30 seeds) of each transformant line was measured using ImageJ software (RRID:SCR_003070). 

Quantification of total chlorophyll was conducted according to [60]. In brief, 100 milligrams (mg) of ground leaf material was incubated in 1.0 mL of 80% (*v/v*) acetone in the dark for 24 h at room temperature. Post incubation, the ground leaf material was pelleted by centrifugation at 15,000× *g* for 5 minutes (min) at room temperature. The absorbance (A) of the resulting supernatant was measured at 646 and 663 nanometers (nm) in a GENESYS 10S spectrophotometer (ThermoFisher Scientific, Sydney, Australia). The total chlorophyll content was calculated by use of the equation: Total chlorophyll (mg/g fresh weight (FW)) = (6.43 × A663 + 18.43 × A646) / sample weight (g) × 1000, according to [56]. The quantification of the abundance of anthocyanin was conducted by incubating 100 mg of freshly ground leaf material in 1.0 mL of acidic methanol (that contained 1% *v/v* HCl) for 2 h at 4 °C. Post-incubation, the ground leaf material was pelleted by centrifugation at 15,000× *g* for 5 min at room temperature. The absorbance of the resulting supernatant was measured at 530 and 657 nm, and the anthocyanin content determined by use of the equation: Anthocyanin (µg/g FW) = (A530 − 0.25 × A657) / sample weight (g) [61].

### 4.3. Histochemical Staining of the Primary Inflorescence Stem of MicroRNA397 Over-Accumulation Transformant Lines

Toluidine blue staining was conducted by incubating free hand-cut cross-sections of the primary inflorescence stem in 0.02% (*w*/*v*) toluidine blue staining solution for 1 min at room temperature, followed by multiple rounds of washing with distilled water. Images of the stained cross-sections were taken using a Leica Fluo III fluorescence microscope (Leica, Wetzlar, Germany).

Phloroglucinol-HCl staining was conducted by incubating free hand-cut cross-sections of the primary inflorescence stem in freshly prepared 1.0% (*w*/*v*) phloroglucinol solution for 2 min at room temperature. The phloroglucinol solution was removed, and the color of stained samples was developed via the addition of 20% (*v*/*v*) HCl and incubation for 2 min at room temperature. Stained cross-sections were mounted on glass slides along with a drop of mounting solution (50% glycerol, 25% lactic acid, 20% HCl, 1.0% phloroglucinol solution, in a *v/v* ratio of 50:40:7:3), and images were taken using a Leica Fluo III fluorescence microscope (Leica, Wetzlar, Germany).

### 4.4. Determination of Total Lignin Content and the Localation of Its Deposition in Arabidopsis Transformant Lines

An acetyl bromide-based protocol according to [31], with some minor modifications for optimization, was utilized to determine total lignin content of the selected *AtMIR397* and *SvMIR397* transformant lines. The total lignin content was measured for inflorescence stem and rosette leaf tissue with 6 to 7 individual plants pooled to produce enough material for 3 replicates of each tissue type and plant line analyzed. Five milligrams of isolated plant cell wall material was dissolved in 1.0 mL of freshly prepared acetyl bromide solution (25% *v/v* acetyl bromide in 100% glacial acetic acid) at 50°C for 2 h. The reaction volume was diluted in 5.0 mL of 100% glacial acetic acid. For each sample, the absorbance of 1.0 mL of acetyl bromide/lignin solution (400 μL of 2.0 M sodium hydroxide, 300 μL of 0.5 M hydroxylamine hydrochloride, and 300 μL of sample solution) was determined at 280 nm in a UV light-specific cuvette using a spectrophotometer (ThermoFisher Scientific, Sydney, Australia). The total lignin content (mg/g dry weight cell wall) was calculated using the equation: Total lignin = (A280/*ε* × L) × (D/S) × 1000, where *ε* is extinction coefficient of *Arabidopsis* (15.69 g^−1^ L cm^−1^), L is the spectrophotometer path length in centimeters (cm), D is the dilution factor of the sample, and S is the weight of the sample in milligrams (mg). 

### 4.5. Bioinformatic Identification of the Setaria viridis MicroRNA397 Small RNA and Its LACCASE Target Genes

The sequence of the mature *S. viridis* miR397 sRNA was identified by using the corresponding sequence of the *Arabidopsis* miR397 sRNA (5′-UCAUUGAGUGCAUCGUUGAUG-3′) as the query to BLASTn (Basic Local Alignment Search Tool for nucleotides) search the *S. viridis* transcriptome (Figure S1). The secondary structure of the putative *SvMIR397* precursor transcript was predicted using the RNAfold Web Server (PRID: SCR_08550) to confirm that the precursor transcript could indeed “fold-up” to form the required stem-loop structure of a miRNA precursor transcript. Post this confirmation, BLASTn was again used to determine which of the two *Arabidopsis* miRNA precursor transcripts (*PRE-MIR397A* and *PRE-MIR397B*) the *S. viridis*-identified miR397 sRNA harboring transcript sequence returned the highest degree of homology to. This analysis revealed the *S. viridis* miR397-harboring transcript sequence to return the highest degree of homology to the *AtPRE-MIR397B* precursor transcript and was therefore named *SvPRE-MIR397B*. The putative target site of the *SvMIR397* sRNA within the coding sequence of each *Arabidopsis* or *S. viridis LAC* transcript was identified using the pSRNATarget (RRID: SCR_013321) and Clustal-Omega tools (RRID: SCR_001591).

### 4.6. Extraction of Total RNA and the Synthesis of Complementary DNA

Total RNA was extracted from the harvested plant material of four biological replicates for wild-type *Arabidopsis* and each analyzed transformant line using TRIzol™ Reagent (ThermoFisher Scientific, Sydney, Australia) according to the manufacturer’s instructions. For the removal of any contaminating genomic DNA, we treated total RNA extracts with the TURBO DNA-free kit as per the manufacturer’s protocol (ThermoFisher Scientific, Sydney, Australia), and then this DNA-free preparation of total RNA was column purified using a RNeasy Plant Mini kit according to the manufacturer’s instructions (Qiagen, Melbourne, Australia).

For the synthesis of first strand complementary DNA (cDNA), we used 1 microgram (1.0 μg) of DNase-treated, column purified total RNA as the template along with Protoscript II Reverse Transcriptase (New England BioLabs, Melbourne, Australia) and an oligo dT_23_ primer (New England BioLabs, Melbourne, Australia), according to the manufacturer’s instructions. A miR397-specific cDNA was synthesized from 300 ng of DNase-treated total RNA using 50 U Protoscript II Reverse Transcriptase (New England BioLabs, Melbourne, Australia) and 50 nanomolar (nM) of miR397-specific stem-loop primer (Table S1). This procedure was also performed for the synthesis of *snoR101*- and *U6*-specific cDNAs using sRNA-specific stem-loop primers (Table S1).

### 4.7. Quantitative Reverse Transcriptase Polymerase Chain Reaction Assessment of MicroRNA397 Abundance and the Expression Level of the LACCASE Target Genes of this Small RNA

All primers used in this study were designed using the Primer3web (RRID: SCR_003139) and NCBI Primer-BLAST (RRID: SCR_003095) online tools and are listed in Table S1. The *UBIQUITIN10* (*UBI10*; *AT4G05320*) and *ELONGATION FACTOR1*α (*EF1*α; *AT5G60390*) transcripts were used as the reference genes to normalize RT-qPCR-generated gene expression data. The sRNAs snoR101 and U6 were used as reference sequences to normalize the quantified abundance of the miR397 sRNA after RT-qPCR. All RT-qPCR reactions were carried out on a Rotor-Gene Q machine (Qiagen, Melbourne, Australia) using GoTaq qPCR Master Mix (Promega, Sydney, Australia), and each reaction contained 1X GoTaq qPCR Master mix, 1.0 μM of each primer, and 50 ng of cDNA template in a 10 μL total reaction volume. The cycling program for product amplification was 1 cycle of 95°C for 10 min, followed by 40 cycles of 95 °C for 15 s and 60 °C for 40 s. The melt curve was generated for each primer pair across the temperature range of 72 to 95 °C, with a temperature increment of 1.0 °C each 5 s period. Each primer pair was assessed using 4 biological replicates, and 3 technical replicates were performed per biological replicate. The resulting RT-qPCR data was analyzed by application of the delta delta Ct (^ΔΔ^Ct) method [62].

## Supplementary Materials

Supplementary Materials can be found at https://www.mdpi.com/1422-0067/21/21/7879/s1. Figure S1: Identification of precursor miR397 sRNA of Setaria viridis and its binding sites on LAC genes. Figure S2: Schematic representation of the pMIR397-OE plant expression vectors. Table S1: List of primers used in RT-qPCR analyses.
