# Supplementary material for: Molecular Manipulation of MicroRNA397 Abundance Influences the Development and Salt Stress Response of Arabidopsis thaliana
Source: Int J Mol Sci. 2020 Oct 23;21(21):7879. doi: 10.3390/ijms21217879 (PMC7660671; doi:10.3390/ijms21217879)
Supplement: Supplementary file 1 [file ijms-21-07879-s001.pdf]

Supplementary Materials

Molecular manipulation of miR397 abundance influences the development and salt stress response of *Arabidopsis thaliana*

Duc Quan Nguyen[1], Christopher W. Brown[1], Joseph L. Pegler[1], Andrew L. Eamens[1*†], Christopher P. L. Grof[1†]

[1] Centre for Plant Science, School of Environmental and Life Sciences, University of Newcastle, Callaghan NSW 2308, Australia; Ducquan.Nguyen@uon.edu.au; Christopher.Brown@uon.edu.au; Joseph.Pegler@uon.edu.au; Chris.Grof@newcastle.edu.au; Andy.Eamens@newcastle.edu.au.

[*] **Correspondence:** Andy.Eamens@newcastle.edu.au; Tel: (+61)249217784.

[†] These authors contributed equally to this work

Received: date; Accepted: date; Published: date

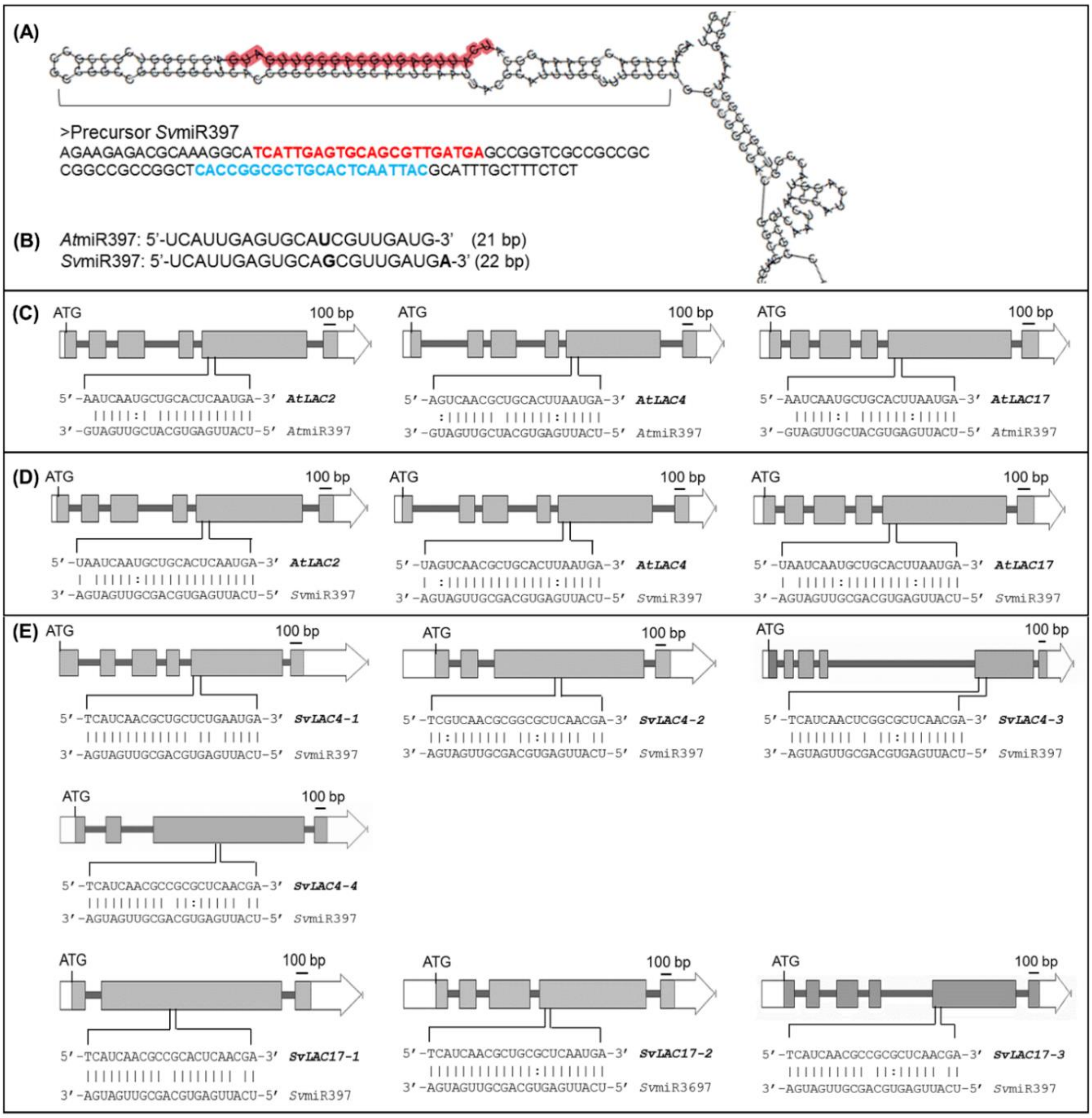

Figure A1: Identification of precursor miR397 sRNA of *Setaria viridis* and its target binding site on *S. viridis LAC4* (*SvLAC4)* and *SvLAC17* genes **(A)** Precursor *S. viridis* miR397 (*Sv*miR397) sRNA sequence was found on an expressed sequence tag (EST) from chromosome 1. Secondary structure of *Sv*miR397 sRNA was predicted by RNAfold online tool, and the mature *Sv*miR397 was predicted based on parameters defined by [1] and [2]. *Sv*miR397 strand has been highlighted in red and *Sv*miR397* strand has been highlighted in blue. **(B)** *Sv*miR397 sRNA is 1 nucleotide longer than *At*miR397 and has 1 mismatched nucleotide at the 13th position. **(C)** Experimentally validated *At*miR397 target sites of *Arabidopsis LAC* genes (*AtLAC*), *AtLAC2, AtLAC4* and *AtLAC17* [3]. **(D)** Predicted *Sv*miR397 target site positions on the *AtLAC2, AtLAC4* and At*LAC17* as determined by psRNATarget and Clustal-Omega online tools. **(E)** Predicted *Sv*miR397 target site positions on the *SvLAC4* and *SvLAC17* as determined by psRNATarget and Clustal-Omega online tools. The white boxes and arrows represent untranslated regions (UTRs), grey shaded boxes represent exons and the dark grey line connecting the grey boxes represents introns. Vertical dashes represent Watson-Crick base pairs and semicolon symbols represent G:U wobble base pairs.

1. Axtell M. J. and Meyers B. C. Revisiting criteria for plant microRNA annotation in the era of big data. *Plant Cell.* **2018**, 30, 272-284.
2. Meyers B. C.; Axtell M. J.; Bartel B.; Bartel D. P.; Baulcombe D.; Bowman J. L.; Cao X.; Carrington J. C.; Chen X.; Green P. J.; Griffiths-Jones S.; Jacobsen S. E.; Mallory A. C.; Martienssen R. A.; Poethig R. S.; Qi Y.; Vaucheret

H.; Voinnet O.; Watanabe Y.; Weigel D. and Zhu J. K. Criteria for annotation of plant MicroRNAs. *Plant Cell.* **2008**, 20, 3186-3190.
3. Abdel-Ghany S. E. and Pilon M. MicroRNA-mediated systemic down-regulation of copper protein expression in response to low copper availability in *Arabidopsis*. *J. Biol. Chem.* **2008**, 283, 15932-15945.

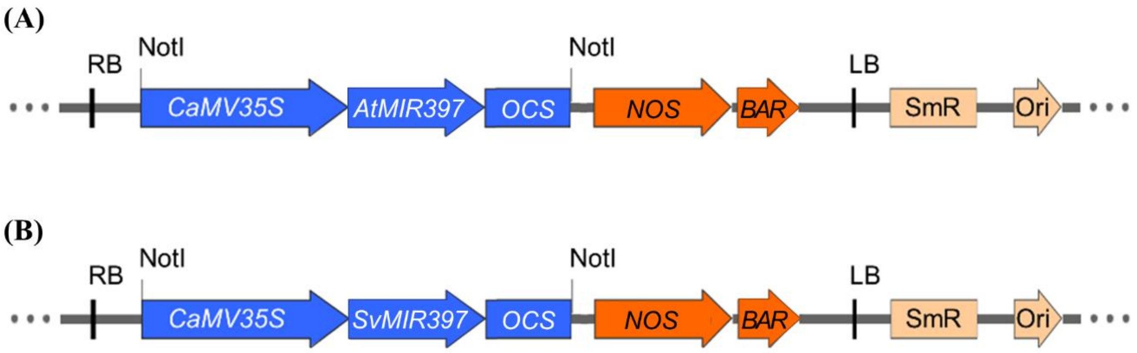

Figure A2: Schematic representation of the *pMIR397*-OE plant expression vectors. The endogenous *MIR397* encoding sequence (genomic sequence encoding the *PRE-MIR397B* was used) of (**A**) *Arabidopsis* and (**B**) *Setaria viridis* was separately inserted between the *CaMV35S* (*Cauliflower mosaic virus* 35S) promoter and the *OT* (*Agrobacterium tumefaciens Octopine synthase*) terminator. *NOS*, *Nopaline synthase* promoter. *BAR*, phosphinothricin (PPT) resistance selection marker; RB, right border; LB, left border; *SmR*, spectinomycin resistance gene; Ori, *E. coli* origin of replication.

Table A1: List of primers used in RT-qPCR analyses

No.	Gene acronyms	Gene ID	Primers	cDNA amplicon	gDNA amplicon
1	***UBQ10***	*AT4G05320*	F: GCCTTGTATAATCCCTGATGAATAAG<br>R: AAAGAGATAACAGGAACGGAAACATAGT	60 bp	60 bp
2	***EF1***	*AT1G07920*	F: TGAGCACGCTCTTCTTGCTTTCA<br>R: GGTGGTGGCATCCATCTTGTTACA	76 bp	76 bp
3	***AtLAC2***	*AT2G29130*	F: CGCTTCCTTGCCGATAATCC<br>R: ACCGTCCAAAACAACCCAAG	99 bp	253 bp
4	***AtLAC4***	*AT2G38080*	F: TCCTCCCCAACAATCCTCAC<br>R: GGGACAAGAGCAGGGTACTT	113 bp	113 bp
5	***AtLAC17***	*AT5G60020*	F: CGACCCGAACAAGGATCCTA<br>R: ATCGAATAGCAGCCCATCCA	95 bp	95 bp
6	***P5CS1***	*AT2G39800*	F: GTTTTTGAATCCCGACCTGA<br>R: TTACCCCCAACAGTCTCTGG	153 bp	153 bp
7	***U6***		SL-RT: GTGCAGGGTCCGAGGTTTT<br>GGACCATTTCTCGAT<br>F: GGAACGATACAGAGAAGATTAGCA<br>R: GTGCAGGGTCCGAGGT	78 bp	78 bp
8	***SNOR101***		F: CTTCACAGGTAAGTTCGCTTG<br>R: AGCATCAGCAGACCAGTAGTT	68 bp	68 bp
9	***AtMIR397***		SL-RT: TCGTATCCAGTGCAGGGTCCG<br>AGGTATTCGCACTGGATACGACCATCAA<br>F: CGCCGGTCATTGAGTGCAGC<br>R: CCAGTGCAGGGTCCGAGGTA	59 bp	59 bp
10	***SvMIR397***		SL-RT: GTCGTATCCAGTGCAGGGTCCG<br>AGGTATTCGCACTGGATACGACTTCATCA<br>F: GCCGGCTCATTGAGTGCAGC<br>R: CCAGTGCAGGGTCCGAGGTA	60 bp	60 bp
